# Psychological Intervention to Promote Resilience in Nurses: A Systematic Review and Meta-Analysis

**DOI:** 10.3390/healthcare12010073

**Published:** 2023-12-28

**Authors:** Suk-Jung Han, Young-Ran Yeun

**Affiliations:** 1 College of Nursing, Sahmyook University, Seoul 01795, Republic of Korea; hansj@syu.ac.kr; 2 College of Nursing, Kangwon National University, Samcheok 25649, Republic of Korea

**Keywords:** nurses, resilience, meta-analysis, psychological intervention

## Abstract

Nurses primarily focus on caring for others, but they also require care and support to enhance their own resilience. Thus, this study aims to determine the effects of psychological interventions on nurses’ resilience support and to define the influence of moderating variables that can affect these effects. The literature search was conducted in 10 electronic databases, and 5 randomized controlled trials and 10 non-randomized controlled trials were finally selected for analysis (a total of 852 participants). Statistical analyses of the effect sizes and homogeneity of the intervention programs were conducted using RevMan 5.3 from the Cochrane Library and the R program. Publication bias in the retrieved studies was tested using contour-enhanced funnel plots. The meta-analysis found that psychological interventions were effective in improving nurses’ resilience immediately after the intervention (SMD = 0.59, 95% CI 0.31 to 0.86, Z = 4.18, *p* < 0.001) and in the short term within three months (SMD = 1.52, 95% CI 0.74 to 2.31, Z = 3.80, *p* < 0.001). Interventions using emotion regulation, relaxation, and self-compassion were particularly effective, and the intervention period was effective in both a short period of 1 day and a long period of more than 12 weeks. In addition, the optimal one-session time was 121–150 min, and offline interventions were more effective than online interventions. Furthermore, the effect size was affected by the intervention time for one session (QB = 12.02, df = 3, *p* = 0.007) and the on/offline intervention method (QB = 5.85, df = 1, *p* = 0.015). These findings may inform the development of targeted interventions and resilience support systems for nurses. However, in the future, more rigorous studies, such as randomized controlled trials, should be conducted to ensure strict control over the variables and to establish a stronger evidence base for the effectiveness of these interventions.

## 1. Introduction

Over the past two decades, new viruses have continued to threaten humanity, including Severe Acute Respiratory Syndrome (SARS), Middle East Respiratory Syndrome (MERS), Ebola virus disease, and now COVID-19 [1].

During the COVID-19 pandemic, healthcare workers experienced anxiety (34.4%), depression (31.8%), stress (40.3%), posttraumatic stress syndrome (11.4%), insomnia (27.8%), psychological distress (46.1%), and burnout (37.4%) [2], and suicide rates increased [3].

In a qualitative meta-synthesis of frontline healthcare workers’ experiences of caring for patients [4], the most common fear expressed by the study participants was fear for their own physical safety, stemming from a deep concern about spreading the infection to patients, colleagues, and family members due to their own infection. They also reported a heightened awareness of the emotional impact of caring for COVID-19 patients over the long term, with sleep disturbance, intrusive memories, constant arousal, and difficulty adjusting to home and daily tasks, even after the peak of the pandemic.

Previous research on the psychological impact on healthcare workers during previous infectious disease outbreaks and successful interventions to manage it [1] suggests that psychological distress among healthcare workers during a viral outbreak is predictable, given the need to care for large numbers of potentially infected patients, and can be mitigated by rapid implementation of effective interventions. In particular, psychological interventions tailored to individual needs have been proposed as a way to reduce the risk of negative psychological outcomes [5,6,7].

Studies on the psychological needs of nurses caring for COVID-19 patients [5] found that even in a difficult environment, the mere presence of mental health support arranged by the hospital organization helped to alleviate nurses’ anxiety. Based on these findings, they suggested forming a psychological task force with the help of nursing management and psychological experts and establishing a psychological support platform to protect nurses’ mental health [8]. Aiello et al. [9] designed and implemented resilience training to maintain the health of individuals within an organization and to protect the organization’s ability to respond to emergencies in the face of an anticipated influenza pandemic. The goal was to increase employee resilience by providing training to prevent and reduce pandemic-related stress. Results showed that 35% of the subjects felt prepared to deal with the pandemic with confidence before the intervention, which increased to 76% after the sessions. Building nurses’ resilience is considered an important intervention to address nurses’ emotional dissonance and mitigate the adverse effects of stressors on their biopsychosocial well-being [10].

Resilience is the ability to transform difficult situations, adversity, stress, and pain in one’s life into experiences of growth and moving forward [11]. It is also reported to be a factor in reducing nurse burnout as an individual’s inner ability to cope flexibly in difficult work environments that cause stress due to heavy workloads [12]. There is a strong association between burnout and resilience [13], and nurses with high resilience are protected from burnout [12].

Resilience is a concept derived from individual differences in human responses to stress and is a psychosocial ability of individuals, developed out of an interest in the fact that not all responses of individuals to stressful situations are negative, but rather that they utilize their strengths and exhibit positive adaptive behaviors to reach or exceed pre-stress levels [14].

A meta-analysis of variables related to resilience in Korean nurses, categorized into protective factors and risk factors, found that resilience was negatively associated with burnout, with the largest effect size, and negatively associated with turnover intention, with a medium effect size [15]. Higher resilience was associated with lower emotional burnout and depression [16], higher levels of stress coping, mental health [17], and psychological well-being [18], and was a significant predictor of secondary traumatic stress [19] and the only significant predictor of posttraumatic growth [20,21].

Nurses spend most of their time caring for others, but they should also engage in self-care to improve their own resilience [22]. Leaders of nursing organizations should strengthen individual and organizational resilience by implementing managerial interventions, such as training in resilience skills and behaviors, as preventive support for nurses who may experience burnout due to high levels of anxiety, stress, and heavy workloads [13,23]. These effective strategies will maintain the mental and psychological health of nurses, strengthening them to stay rather than choose turnover at a time when they are most needed.

Psychological interventions to promote resilience should consider the understanding and needs of the target audience, be adaptable to the local context, and select appropriate interventions that evenly enhance the attributes of the subfactors of resilience [24].

The previous study conducted a meta-analysis by selecting only descriptive correlational articles on the relationship between resilience and related variables in Korean nurses [15].

Therefore, this study aimed to conduct a systematic review and meta-analysis by selecting experimental studies that applied various interventions to affect nurses’ resilience not only in Korea but also globally.

## 2. Materials and Methods

### 2.1. Eligibility Criteria

This study was conducted according to the Preferred Reporting Items for Systematic Reviews and Meta-Analysis (PRISMA) guidelines [25]. The inclusion and exclusion criteria were set according to the key questions, and the literature was selected accordingly. The protocol for this review has been registered in the International Prospective Register for Systematic Reviews (PROSPERO; registration ID: CRD42023423670).

All articles written in English or Korean were selected by specifying the inclusion and exclusion criteria centered on the Participant, Intervention, Comparison, Outcome study design (PICO-SD).

The research questions that guided the design of this study were: “What is the content of a resilience promotion program for nurses?” and “How does a resilience promotion program provided to nurses affect their resilience?”.

These included studies that applied psychological interventions (I) to nurses (P). Regardless of where the intervention was delivered, we included studies that provided individual or group interventions for nurses. We also included studies with a control group (C) that did not provide resilience-enhancing psychological intervention or a comparison group that provided general education or counseling. However, single-arm studies with no comparison groups were excluded. For outcome (O), we selected studies that had a value for the resilience outcome measured after the psychological intervention for nurses and presented the mean and standard deviation of the outcome measured after participation in the program so that we could calculate the effect size for the experimental and control groups.

### 2.2. Search Strategy

The literature search was conducted in January 2023, and there was no limited publication period. In addition to the search terms, we limited the research design to experimental studies involving humans, limited the languages to Korean and English, and included journal articles, theses, and dissertations to reduce publication bias. A bibliographic management program (EndNote 20) was used to classify the studies and remove duplicate articles.

For the literature search, we used PubMed, Embase, CINAHL, PsycInfo, Cochrane, RISS, KISS, KMbase, Science On, and KoreaMed among domestic and international specialized search data. The researchers checked whether each keyword was present in the title and abstract and whether the study met the inclusion criteria and reviewed the full text of the final article before inclusion in the analysis. All processes for selecting the retrieved articles were performed independently by two researchers, and in cases of disagreement, the final articles were selected through discussion.

For the literature search in international databases, the search formula used both Medical Subject Headings (MeSHs) and the Life Sciences Terminology Index (Emtree) and utilized the BLIEN operator to apply AND/OR and truncation searches to search for significant terms together, which can increase the sensitivity and specificity of the search. The main search terms were “nurses” AND “resilience OR resilien*”, “therapy” OR “Counseling” OR “Training Support” OR “Teaching” OR “Learning” OR “OR” (Appendix A). The domestic database search was based on the search strategy used for the international search and was conducted using the following conceptual terms: “nurse”, “recovery”, “overcoming”, “resilience”, “program”, “intervention”, “education”, “protocol”, “treatment”, “development”, “training”, “counseling”, “coaching”, and “promotion”.

### 2.3. Risk of Bias Assessment

The quality of the literature included in the meta-analysis was assessed for intention-to-treat using the Revised Cochrane risk of bias for randomized trials (RoB2) [26]. RoB2, used to assess bias in RCT studies, consists of items to assess the risk of bias in five areas: randomization process, deviation from the intended intervention, missing data, outcome measures, selection of reported outcomes, and overall risk of bias in the study.

For non-randomized controlled trials (NRCT), the Risk of Bias Assessment Tool for Non-Randomized Studies 2.0 (RoBANS 2.0) was used. RoBANS 2.0 covers the following eight domains: comparison possibility of participants, selection of participants, confounding variables, measurement of exposure, blinding of the outcome assessments, outcome evaluation, incomplete outcome data, and selective outcome reporting [27]. Two researchers independently assessed the final selected articles, and disagreements were resolved through discussion.

### 2.4. Data Analysis

Statistical analyses of effect size and homogeneity of intervention programs were conducted using RevMan 5.3 from the Cochrane Library and the R 4.2.3 program. The Standard Mean Difference (SMD) was selected for the effect size calculation. Higgins’ I^2^ test was used to assess the statistical homogeneity of effect sizes [28]. If homogeneity was found, the effect sizes were merged using a fixed-effects model; if heterogeneity was found, the effect sizes were calculated using a random-effects model. The statistical significance of the effect size was determined using the overall effect test and 95% confidence interval (CI) with a significance level of less than 5%. The effect size was interpreted according to Cohen’s interpretation criteria [29]. If there were two experimental groups in a study, we analyzed each group as a separate study based on previous research [30]. A meta-ANOVA was conducted to directly test for differences in effect sizes between the subgroups. Publication bias in the retrieved studies was tested using a contour-enhanced funnel plot.

### 2.5. Ethical Consideration

This study was approved by the Internal Review Board of Sahmyook University (IRB No. SYU 2022-10-002). The data were collected from published articles in web-based databases, and no harm or risk was posed by the research to the participants. In addition, the collected data will not be used for any purpose other than for this study.

## 3. Results

### 3.1. Included Studies

**Figure 1:**
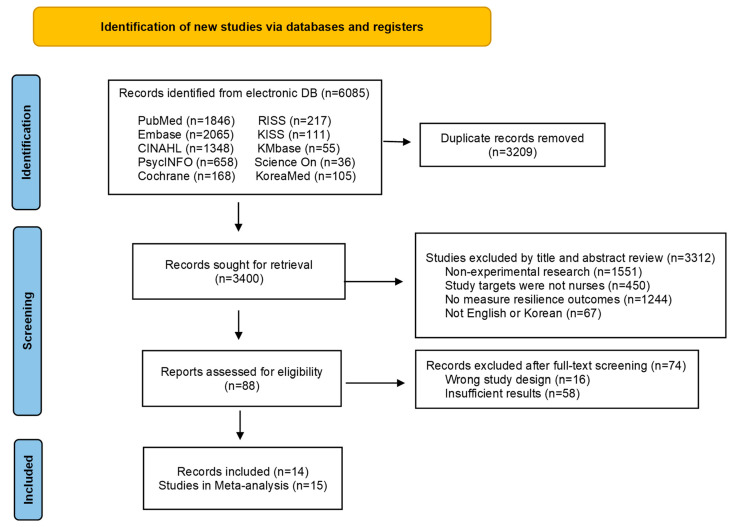
PRISMA flow diagram.

A total of 6609 articles were retrieved from the database. After excluding 3209 duplicate searches, 88 articles were selected for the first round by reviewing the titles and abstracts of 3400 articles. Fourteen articles were selected after reviewing 88 full texts based on the selection and exclusion criteria, and a meta-analysis was conducted on 15 studies (Figure 1). In the study by Hsieh et al. [31], the group that performed biofeedback training (Hsieh 2020 A) and the group that performed smartphone-delivered biofeedback training (Hsieh 2020 B) were compared with the control group. The entire process of selecting the literature was independently conducted by two researchers, and in case of disagreement, the final paper was selected through discussion.

### 3.2. Risk of Bias Assessment

**Figure 2:**
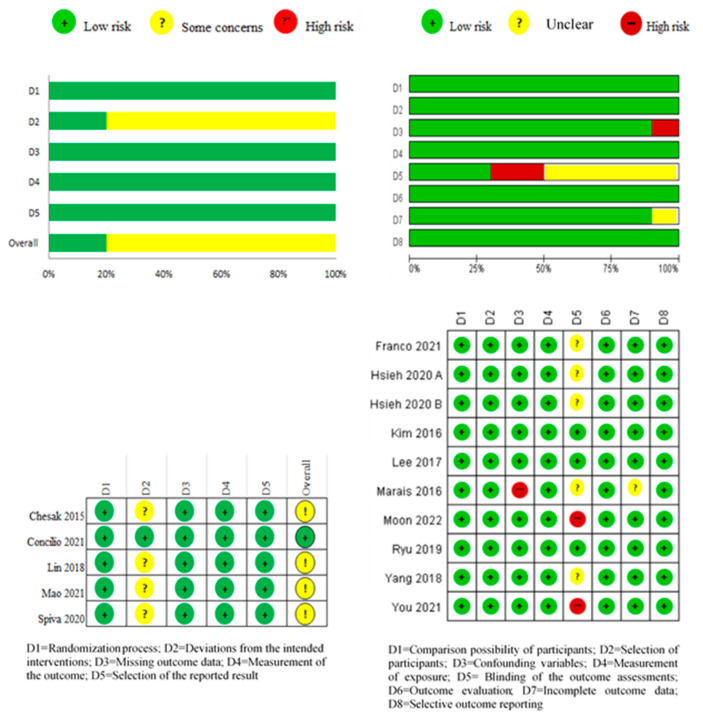
Assessment of risk of bias. Assessment of risk of bias was performed using Risk of Bias 2 (RoB 2) for randomized controlled trials and Risk of Bias Assessment Tool for Non-Randomized Studies 2.0 (RoBANS 2.0) for non-randomized controlled trials [29,30,31,32,33,34,35,36,37,38,39,40,41,42].

The risk of bias assessment results for the five RCTs are shown in Figure 2. As for deviations from the intended interventions, in the D2 domain, there was some concern in four out of five trials (80%), and all other domains were rated as low risk. As a result of the overall risk of bias assessment, only one study by Concilio et al. [32] was evaluated as low-risk, and all others were evaluated as concerning. Studies by Chesak et al. [33], Lin et al. [34], Mao et al. [35], and Spiva et al. [36] showed a dropout rate of 19% to 30% in the experimental group, which was evaluated as a risk of bias.

The results of the risk of bias assessment for the NRCTs are shown in Figure 2. The risk of bias was low in other domains, except for blinding of the outcome assessments. In blinding of the outcome assessments, two out of ten studies (20.0%) were at high risk of bias because the researcher conducted the direct evaluation, five studies (50.0%) did not mention the evaluator, and three studies (30.0%) were performed using the appropriate evaluator’s blinding.

### 3.3. Characteristics of the Included Studies

The characteristics of the 15 studies (14 articles) included in the meta-analysis are shown in Table 1. Regarding the study design, there were five RCTs and ten NRCTs, all of which were published after 2015. There were 10 studies (66.7%) conducted in Asia, 6 (40.0%) in Korea, 2 (13.3%) in China, and 2 (13.3%) in Taiwan. Other than that, there were 4 (26.7%) in the USA and 1 (6.7%) in South Africa. The total number of participants was 852, and the experimental and control groups were 420 and 432, respectively. For setting, nine studies (60.0%) were conducted on all nurses in hospitals, two (13.3%) on psychiatric wards, two (13.3%) on emergency room studies, one (6.7%) on pediatric units, and one (6.7%) on intensive care units. Clinical nurses were the most common research subjects with 5 studies (33.3%), and the average age was 20, with 7 studies (46.7%). The program content varied from cognitive reframing to social support. As for the duration of intervention, 6 to 8 weeks was the most frequent, with 6 studies (40.0%), and the time per session was less than 120 min, with 6 studies (40.0%) being the most. Ten studies (66.7%) applied interventions as a group, and more studies were conducted offline (12 studies, 80.0%) than online (3 studies, 20.0%).

### 3.4. Meta-Analysis

The analysis of 15 studies reporting post-intervention results showed a moderate level of heterogeneity, with a Higgins I^2^ = 72%. The calculated effect size was 0.59 (95% CI 0.31 to 0.86), indicating a statistically significant difference (Z = 4.18, *p* < 0.001). The examination of five studies reporting follow-up results 4 weeks to 3 months after the end of the intervention showed a high level of heterogeneity, with a Higgins I^2^ = 88%. The calculated effect size was 1.52 (95% CI 0.74 to 2.31), indicating a statistically significant difference (Z = 3.80, *p* < 0.001).

To determine the cause of heterogeneity, a subgroup analysis was performed on the studies that reported post-intervention results. The subgroup analysis was conducted by dividing the intervention content by the duration, time per session, and method.

#### 3.4.1. Comparison of Effects According to Intervention Contents

**Figure 3:**
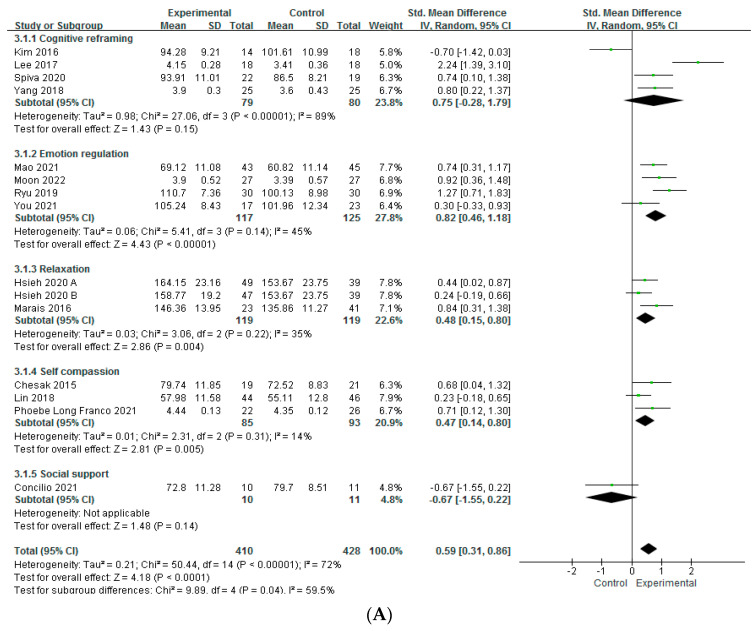

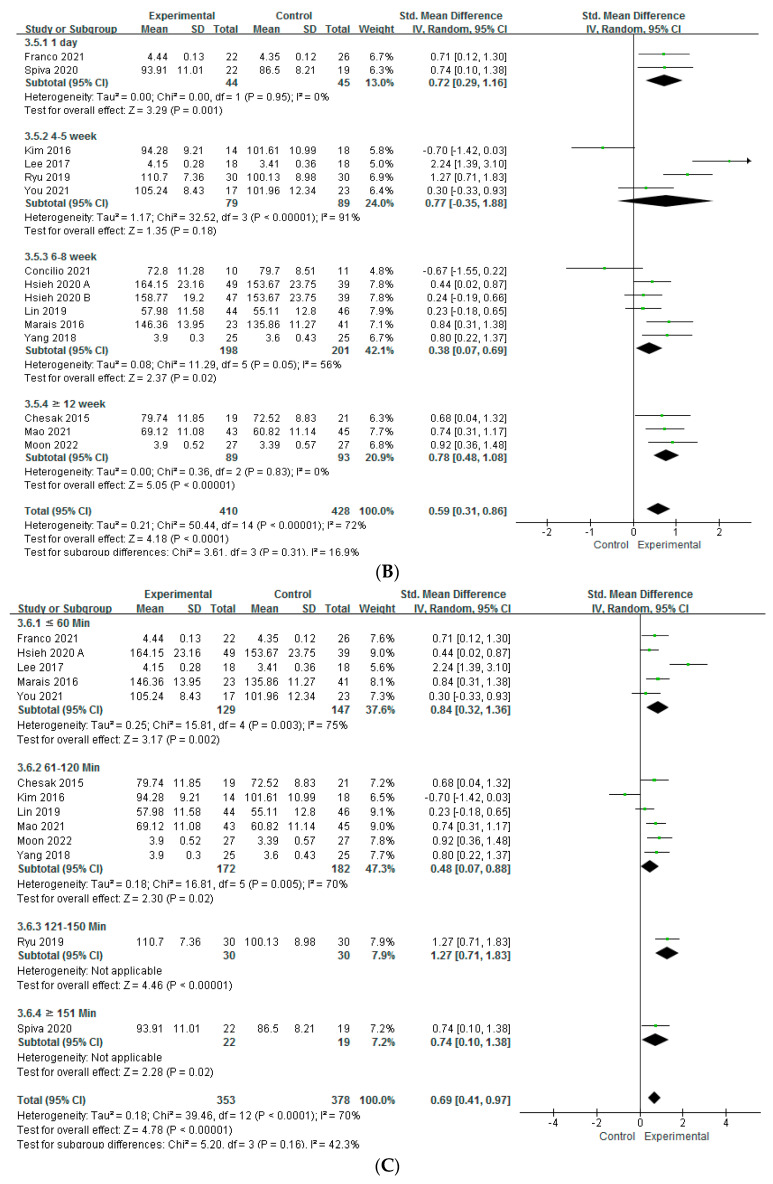

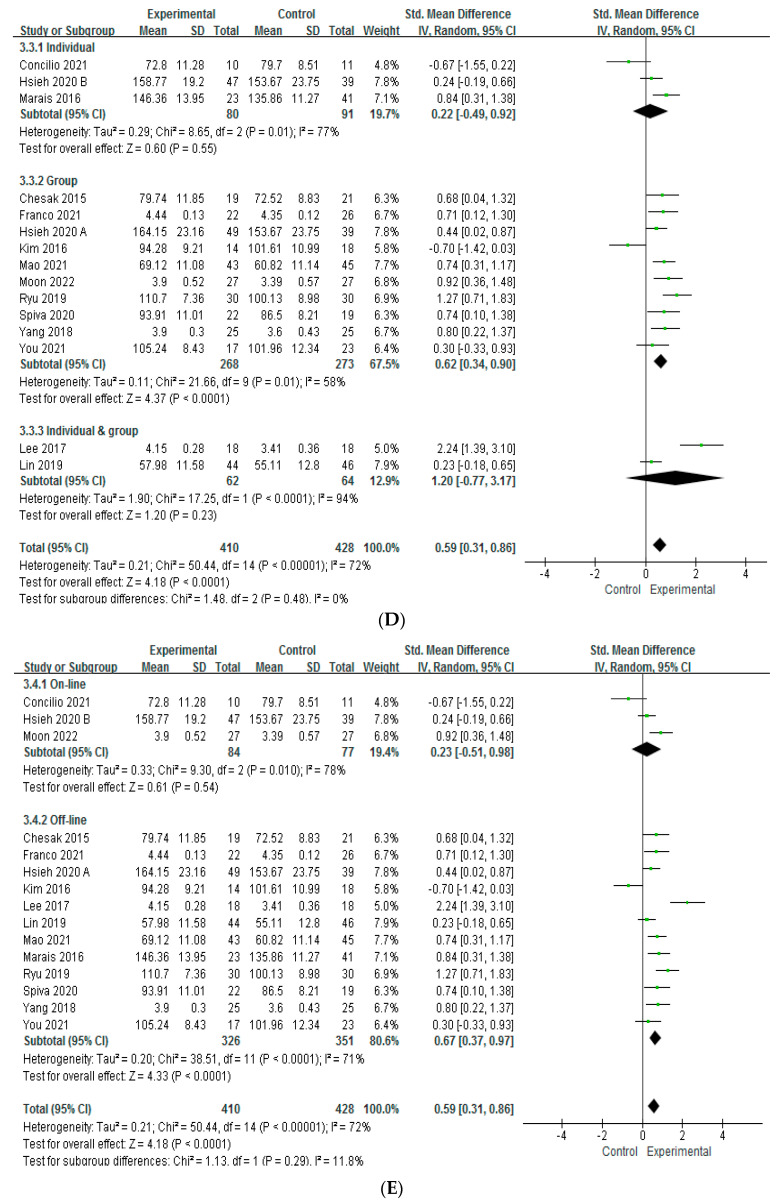
(**A**) Forest plot of the effects of psychological intervention on resilience according to intervention contents [29,30,31,32,33,34,35,36,37,39,40,41,42]. (**B**) Forest plot of the effects of psychological intervention on resilience according to intervention duration [29,30,31,32,33,34,35,36,37,38,39,40,41,42]. (**C**) Forest plot of the effects of psychological intervention on resilience according to intervention time per session [29,30,31,33,34,35,36,37,38,39,40,41,42]. (**D**). Forest plot of the effects of psychological intervention on resilience according to individual/group [29,30,31,32,33,34,35,36,37,38,39,40,41,42]. (**E**). Forest plot of the effects of psychological intervention on resilience according to online/offline [29,30,31,32,33,34,35,36,37,38,39,40,41,42].

After reviewing the program, the main content was divided into five categories: cognitive reframing, emotion regulation, relaxation, self-compassion, and social support. As a result of the subgroup analysis, heterogeneity (Higgins I^2^ = 89%) occurred only in cognitive reframing, and heterogeneity was resolved in the other groups, so the cognitive reframing group was considered as the cause of heterogeneity. Based on the comparison of the effect sizes, it was found that emotion regulation (SMD = 0.82, 95% CI = 0.46 to 1.18, Z = 4.43, *p* < 0.001), relaxation (SMD = 0.48, 95% CI = 0.15 to 0.80, Z = 2.86, *p* = 0.004), and self-compassion (SMD = 0.47, 95% CI = 0.14 to 0.80, Z = 2.81, *p* = 0.005) were effective in increasing resilience and were statistically significant according to Figure 3A.

#### 3.4.2. Comparison of Effects According to Duration

Only studies with a duration of 4–5 weeks were heterogeneous (Higgins I^2^ = 91%), suggesting that the duration of intervention was considered as a cause of heterogeneity. Studies with duration of 1 day (SMD = 0.72, 95% CI = 0.29 to 1.16, Z = 3.29, *p* = 0.001), studies with a duration of 6–8 weeks (SMD= 0.38, 95% CI = 0.07 to 0.69, Z = 2.37, *p* = 0.02), and studies over 12 weeks (SMD = 0.78, 95% CI = 0.48 to 1.08, Z = 5.05, *p* < 0.001) showed a statistically significant increase in resilience (Figure 3B).

#### 3.4.3. Comparison of Effects According to One-Session Time

Studies with a one-session duration of less than 60 min (Higgins I^2^ = 75%) and 61 to 120 min (Higgins I^2^ = 70%) were heterogeneous (Figure 3C). However, all four categories of session time showed a statistically significant increase in resilience. The SMD and 95% CI were as follows: less than 60 min (SMD = 0.84, 95% CI = 0.32 to 1.36, Z = 3.17, *p* = 0.002), 61–120 min (SMD = 0.48, 95% CI = 0.07 to 0.88, Z = 2.30, *p* = 0.02), 121–150 min (SMD = 1.25, 95% CI = 0.71 to 1.83, Z = 4.46, *p* < 0.001), and more than 151 min (SMD = 0.74, 95% CI = 0.10 to 1.38, Z = 2.28, *p* = 0.02).

#### 3.4.4. Comparison of Effects According to Methods

The effect comparison according to the intervention method was analyzed by dividing them into individual/group and on/offline categories.

1. Comparison of effects according to individual/group

Studies that applied intervention as an individual (Higgins I^2^ = 77%) and those that applied both individuals and groups simultaneously (Higgins I^2^ = 94%) were heterogeneous. Only group-based interventions showed a statistically significant increase in resilience (SMD = 062, 95% CI = 0.34 to 0.90, Z = 4.37, *p* < 0.001) (Figure 3D).

2. Comparison of effects according to both on/offline

Studies conducted online (Higgins I^2^ = 78%) and studies conducted offline (Higgins I^2^ = 71%) were heterogeneous. However, studies conducted offline showed a statistically significant improvement in resilience (SMD = 067, 95% CI = 0.37 to 0.97, Z = 4.33, *p* < 0.001) (Figure 3E).

### 3.5. Moderation Effect Analysis

To better understand the differences in effect size between subgroups and the influence of moderating variables that affect the overall effect size, a meta-ANOVA was conducted. Table 2 presents the results of this study.

3. As a result of comparing the effect size of resilience by program, cognitive reframing was 0.90, emotion regulation 0.78, relaxation 0.19, self-compassion 0.61, and social support −0.66, and there was no statistically significant difference in the effect size between groups (QB = 6.49, df = 4, *p* = 0.165).
4. As a result of comparing the effect size of resilience according to the intervention duration, 1 day was 0.58, 4–5 weeks 0.93, 6–8 weeks 0.28, and ≥12 weeks 0.74, and there was no statistically significant difference in effect size between groups (QB = 2.62, df = 3, *p* = 0.454).
5. As a result of comparing the effect size of resilience according to the intervention one-session time, ≤60 min was 0.39, 60–120 min 0.87, 121–150 min 1.27, and ≥151 min 0.74, and there was a statistically significant difference in effect size between groups (QB = 12.02, df = 3, *p* = 0.007). As a result of the post hoc analysis, the effect size was the largest in the group with 121–150 min of time per session.
6. Among the intervention methods, the effect size of resilience according to individual/group was individual −0.06, group 0.78, and individual and group 0.48, and there was no statistically significant difference in the effect size between the three groups (QB = 4.57, df = 2, *p* = 0.101).
7. Comparing the effect size of resilience between the online group and the offline group showed that the effect size between each group was −0.14 and 0.78, and there was a statistically significant difference in the effect size between the two groups (Q = 5.85, df = 1, *p* = 0.015).

### 3.6. Publication Bias

**Figure 4:**
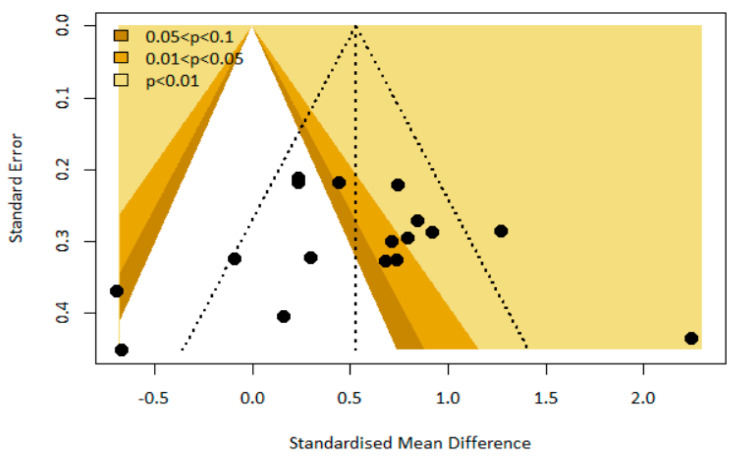
Contour-enhanced funnel plot.

Based on the results obtained using the contour-enhanced funnel plot, it was not possible to conclude that there was a likelihood of publication bias because the *p*-value (*p* = 0.41) by the Begg and Mazumdar test was greater than the significance level of 0.05 (Figure 4).

## 4. Discussion

This study aimed to establish a foundation for developing a tailored, sustainable, and field-specific program to enhance nurses’ resilience by examining the impact of psychological interventions on promoting resilience and identifying the critical program elements that influence the magnitude of the intervention’s effects.

The results of this study found that psychological intervention had a medium effect size in promoting nurses’ resilience post-intervention and a large effect size at the follow-up period of four weeks to three months. This means that the psychological intervention shows a continuous effect not only immediately after the intervention but also for a short period of time (≤3 months), which is considered an important basis for setting up an educational cycle when designing future programs. Zhai et al. [43], in a meta-analysis of 13 studies, also reported that resilience training had a moderate effect size in promoting resilience in nurses, supporting the findings of this study. Joyce et al. [44], in a meta-analysis of 11 RCTs designed to improve individual resilience, found a moderate positive effect of resilience interventions, similar to the findings of this study. Furthermore, Angelopoulou and Panagopoulou [45], who evaluated the effectiveness of an intervention to promote resilience in physicians, found that the intervention was associated with small but significant benefits.

Through analysis of the main contents of a psychological intervention program, it was found that a program focusing on emotion regulation, relaxation, and self-compassion as the main contents led to an improvement in resilience. Among these factors, emotion regulation had the greatest effect on resilience. Kunzler et al. [46] supported the results of this study by reporting relaxation, psychoeducation, emotion regulation, cognitive strategies, problem-solving, and the strengthening of internal and external resources as positive program contents for psychological interventions for nurses. Emotional regulation refers to the process through which individuals manage their emotional experiences and expressions [47]. Emotional regulation interventions, which may include elements such as empathy, self-growth, and positive emotions, can help nurses who experience intense emotional labor to accept their own emotions, understand others’ emotions, and respond positively to negative situations. These interventions are believed to improve nurses’ abilities to recover from emotional stress and maintain their well-being [48,49].

Meanwhile, the effectiveness of resilience intervention strategies may vary depending on the participants and context. In a meta-analysis of 11 RCTs promoting individual resilience, Joyce et al. [44] found that a combination of CBT and mindfulness techniques had a positive impact on individual resilience. Llistosella et al. [50] meta-analyzed the effects of school-based interventions on adolescent resilience, providing evidence that multicomponent and CBT interventions increase resilience in the short term among at-risk early adolescents. Ang et al. [51] meta-analyzed 25 RCTs and concluded that a resilience intervention consisting of skills to improve social competence significantly increased resilience in college students. In an analysis of resilience interventions for physicians, Angelopoulou and Panagopoulou [45] found that emotional–supportive–coping interventions showed greater improvements compared to mindfulness–meditation–relaxation interventions. According to previous studies, those who receive more benefits may vary depending on which of the various resilience factors, such as cognitive flexibility, coping, self-efficacy, self-sufficiency, self-care, or mindfulness, are desired to be improved. The content of an intervention is an important factor that influences how the intervention should be applied. Therefore, when drafting guidelines to promote nurses’ resilience, it is important to carefully plan the content of interventions appropriate for nurses.

Previous studies suggest that who benefits more may depend on which of several resilience factors you want to improve, such as cognitive flexibility, coping, self-efficacy, self-sufficiency, self-care, and mindfulness. The content of the intervention is an important factor that influences how the intervention should be applied. Therefore, when drafting guidelines to promote resilience in nurses, it is important to carefully plan the content of the intervention that is appropriate for nurses based on research.

The sub-analysis examined the relationship between the duration of an intervention and one-session time on the effect size of the intervention. The results showed large effect sizes for interventions that were either short (1 day) or long (more than 12 weeks). Additionally, the largest effect size was observed for interventions with a one-session time of 121–150 min. In the meta-ANOVA post-analysis, the group with a one-session time of 121–150 min showed the largest statistically significant effect size. This finding suggests that interventions with longer session durations may be more effective in producing positive outcomes. According to Yang [37], nurses who participated in resilience training preferred shorter intervention periods and sessions, even if individual sessions were longer. Ryu and Kim [30] also found that integrating the opinions of nursing managers and training head nurses reduced the intervention period to four weeks, with each session lasting two and a half hours. Similarly, Kim and Park [29], who designed a program for emergency room nurses, incorporated the opinion of nurses that it would be difficult to participate in a program lasting more than five weeks. They designed a five-week program with sessions lasting 80 min each. Therefore, it may be beneficial to apply a program with a short intervention period and longer session length, even if it is 121–150 min long, to address time constraints and improve program outcomes for nurses working in special environments with three shifts.

Based on the results of the subfactor analysis, a medium effect size was found only in offline studies conducted between 2015 and 2021. In contrast, online studies conducted between 2000 and 2022 showed a small effect size. The COVID-19 pandemic has caused difficulties in face-to-face interventions but has also led to the activation of digital non-face-to-face methods, such as smartphone applications and Zoom. In a study by Hsieh et al. [31], both face-to-face biofeedback training (BT) and smartphone-delivered BT (SDBT) were effective in improving resilience; however, SDBT was more effective in reducing occupational stress. This is because SDBT can be implemented during off-hours when individuals feel relatively less stressed, whereas BT can only be provided in hospitals where nurses are already stressed. Among non-face-to-face methods, interventions using smartphone applications are particularly efficient in terms of cost, accessibility, and space and are more likely to be implemented by nurses with time constraints [52,53,54]. Further research is required to verify the effectiveness of these interventions in improving resilience.

Through the selection and exploration of moderating variables in a meta-analysis, it was found that there was a statistically significant difference in the effect size of resilience interventions for nurses depending on the one-session duration and on/offline delivery method. Thus, it has been confirmed that intervention time and method are important factors to consider when conducting future research on nurses’ resilience. Careful consideration of these moderating variables is essential for accurately evaluating the effectiveness of resilience interventions for nurses.

This study has several limitations that need to be acknowledged. First, the effect size of the intervention may have been either overestimated or underestimated due to the limited number of randomized controlled trials (RCTs), which can affect the internal validity of this study. Second, the use of various intervention contents and measurement tools by different researchers makes it challenging to identify the intervention factors that show differences in effect sizes through a meta-ANOVA. Inconsistencies in intervention content and assessment tools may also be a source of heterogeneity, so caution is needed in interpreting the results. Third, there is a lack of studies that examined the follow-up effect; thus, studies from four weeks to three months after the intervention were integrated and evaluated. Therefore, it is necessary to re-examine the change after the accumulation of studies that confirm the change in resilience over time after the intervention. Despite these limitations, this study provides a blueprint for developing a customized and sustainable resilience enhancement program for clinical nurses by analyzing changes in resilience and classifying the content, period, time, and method of psychological intervention. In addition, this study not only provides significant insights for the development of effective interventions to enhance the resilience of clinical nurses but also supplies important secondary evidence for future research by carefully examining heterogeneity through meta-analysis.

## 5. Conclusions

This study reviewed various studies on the effectiveness of psychological interventions aimed at improving the resilience of nurses. The results indicated that psychological interventions were effective in enhancing the resilience of nurses not only immediately after the intervention but also for a short period of up to three months. This study provides useful and important secondary evidence to guide the development of resilience promotion programs and future research by carefully examining the heterogeneity of effect sizes through a systematic review and meta-analysis and by identifying intervention times and methods as important factors to consider for improving nurses’ resilience.

## Figures and Tables

**Table 1:** Characteristics of Included Studies.

First Author (Years)	Country	Design	Setting	Exp. (n)	Cont. (n)	Age (M ± SD)	% over Bachelor	Participants	Program	Major Contents	Duration (Week)	Session	Min	Individual/ Group	On/off Line	Scale
Chesak (2015) [33]	USA	RCT	Hospital	19	21	27.9 ± 7.1	89.0	New nurses	Stress management and resiliency training	Self-compassion	12	12	90	Group	Off	CD-RISC
Concilio (2021) [32]	USA	RCT	Hospital	10	10	21–30	81.0	Newly licensed graduate nurses	Social support intervention	Social support	6	24	NR	Individual	On	CD-RISC
Franco (2021) [38]	USA	NRCT	Pediatric ward	22	26	41.9	NR	Clinical or non-clinical nurses	Self-compassion	Self-compassion	1 day	6	60	Group	Off	RADJE
Hsieh A (2020) [31]	Taiwan	NRCT	Psychiatric ward	49	39	38.5 ± 9.2	69.3	Psychiatric ward nurses	Biofeedback training	Relaxation	6	6	60	Group	Off	RS
Hsieh B (2020) [31]	Taiwan	NRCT	Psychiatric ward	47	39	32.2 ± 6.3	82.9	Psychiatric ward nurses	Smartphone-delivered biofeedback training	Relaxation	6	6	NR	Individual	On	RS
Kim (2016) [29]	Korea	NRCT	Emergency room	14	18	27.5 ± 3.4	57.1	Emergency room nurses	Overcoming compassion fatigue	Cognitive reframing	5	5	80	Group	Off	ERK
Lee (2017) [39]	Korea	NRCT	Emergency room	18	18	28.5	NR	Emergency room nurses	Violence coping	Cognitive reframing	4	8	50	Individual and group	Off	PRPS
Lin (2019) [34]	China	RCT	Hospital	44	46	32.8 ± 7.4	63.7	Full-time nurses	Stress reduction	Self-compassion	8	56	120	Individual and group	Off	CD-RISC
Mao (2021) [35]	China	RCT	Hospital	53	50	30.9 ± 5.7	97.1	Clinical nurses	Emotional intelligence training	Emotional regulation	46	48	60~120	Group	Off	CD-RISC
Marais (2016) [40]	South Africa	NRCT	Hospital	23	41	43.0 ± 10.2	NR	OR and ICU nurses	Sensory stimulation therapy	Relaxation	8	NR	30	Individual	Off	RS
Moon (2022) [41]	Korea	NRCT	Long-term care hospital	27	27	43.3 ± 6.2	44.4	Clinical nurses	Resilience	Emotional regulation	12	8	120	Group	On	CD-RISC
Ryu (2020) [30]	Korea	NRCT	Hospital	30	30	26.8 ± 3.5	66.7	Clinical nurses	Emotional coaching	Emotional regulation	4	4	150	Group	Off	RSN
Spiva (2020) [36]	USA	RCT	Hospital	22	19	43.2 ± 9.6	73.7	Charge nurses	Leadership training	Cognitive reframing	1 day	3	480	Group	Off	CD-RISC
Yang (2018) [37]	Korea	NRCT	Hospital	25	25	26.6 ± 2.8	48.0	Clinical nurses	Resilience enhancement	Cognitive reframing	7	7	100	Group	Off	RSN
You (2021) [42]	Korea	NRCT	Intensive care unit	17	23	26.8 ± 3.5	66.7	ICU nurses	Expressive writing	Emotional regulation	5	5	30	Group	Off	RSN

RCT: randomized controlled trials, NRCT: non-randomized controlled trials, NR: not reported, CD-RISC: Connor–Davidson Resilience Scale, RS: Resilience Scale, ERK: Ego-resiliency scale, PRPS: Polk Resilience Patterns Scale, RADJE: Resiliency activation and decompression and job engagement, RSN: Resilience scale for nurses.

**Table 2:** Meta-ANOVA for moderators.

Category		k	SMD	95% CI	Tau^2^	Q_B_	df	*p*
Program	Cognitive reframing	4	0.90	0.32,1.48	0.24	6.49	4	0.165
	Emotion regulation	4	0.78	0.22, 0.35				
	Relaxation	3	0.19	−0.45, 0.84				
	Self-compassion	3	0.61	−0.01, 1.25				
	Social support	1	−0.66	−1.98, 0.65				
Duration	1 day	2	0.58	−0.30, 1.46	0.33	2.62	3	0.454
	4–5 weeks	4	0.93	0.29, 1.57				
	6–8 weeks	6	0.28	−0.24, 0.80				
	≥12 weeks	3	0.74	0.01, 1.48				
One-session time	≤60 min ^a^	5	0.39	0.16, 0.62	0.10	12.02	3	0.007 (a < b < c)
	61–120 min ^b^	6	0.87	0.62, 1.12				
	121–150 min ^c^	1	1.27	0.67, 1.86				
	≥151 min ^ab^	1	0.74	0.07, 1.41				
Individual/group	Individual	3	−0.06	−0.76, 0.63	0.24	4.57	2	0.101
	Group	10	0.78	0.42, 1.14				
	Individual and group	2	0.48	−0.26, 1.23				
On/offline	Online	3	−0.14	−0.79, 0.50	0.19	5.85	1	0.015
	Offline	12	0.73	0.44, 1.03				

## Data Availability

The datasets used and/or analyzed during the current study are available from the corresponding author upon reasonable request.

## Appendix A. Search Strategy to Identify Relevant Trials from PubMed

**Table:**

	Search	Query	Items Found
P	#1 MeSH	“Nurses” [MeSH Terms]	96,403
	#2 Natural Term	“nurse*”[Title/Abstract]	312,476
	#3	#1 OR #2	354,664
I	#4 MeSH	(((((““therapy”“ [Subheading]) OR ““Counseling”“[Mesh]) OR ““Training Support”“[Mesh]) OR ““Teaching”“[Mesh]) OR ““Learning”“[Mesh]) OR ““Education”“[Mesh]	8,921,116
	#5 Natural Term	(((((((((((((((intervention*[Title/Abstract]) OR (program*[Title/Abstract])) OR (therap*[Title/Abstract])) OR (counsel*[Title/Abstract])) OR (coach*[Title/Abstract])) OR (train*[Title/Abstract])) OR (teach*[Title/Abstract])) OR (learn*[Title/Abstract])) OR (educat*[Title/Abstract])) OR (manag*[Title/Abstract])) OR (enhanc*[Title/Abstract])) OR (increas*[Title/Abstract])) OR (develop*[Title/Abstract])) OR (treat*[Title/Abstract])) OR (protocol*[Title/Abstract])) OR (promot*[Title/Abstract]) “““intervention*”“[Title/Abstract] OR ““program*”“[Title/Abstract] OR ““therap*”“[Title/Abstract] OR ““counsel*”“[Title/Abstract] OR ““coach*”“[Title/Abstract] OR ““train*”“[Title/Abstract] OR ““teach*”“[Title/Abstract] OR ““learn*”“[Title/Abstract] OR ““educat*”“[Title/Abstract] OR ““manag*”“[Title/Abstract] OR ““enhanc*”“[Title/Abstract] OR ““increas*”“[Title/Abstract] OR ““develop*”“[Title/Abstract] OR ““treat*”“[Title/Abstract] OR ““protocol*”“[Title/Abstract] OR ““promot*”“[Title/Abstract]	17,671,288
	#6	#4 OR #5	20,521,852
O	#7 MeSH	Resilience, Psychological [MeSH Terms]	8036
	#8 Natural Term	resilience [Title/Abstract]) OR (resilien*[Title/Abstract]	53,035
	#9	#7 OR #8	54,120
	#10	#3 AND #6 AND #10	1846
